# B cell–intrinsic TLR7 expression drives severe lupus in TLR9-deficient mice

**DOI:** 10.1172/jci.insight.172219

**Published:** 2023-08-22

**Authors:** Haylee A. Cosgrove, Sebastien Gingras, Minjung Kim, Sheldon Bastacky, Jeremy S. Tilstra, Mark J. Shlomchik

**Affiliations:** 1 Department of Immunology,; 2 Department of Pathology,; 3 Department of Medicine, and; 4 Lupus Center of Excellence, University of Pittsburgh School of Medicine, Pittsburgh, Pennsylvania, USA.

**Keywords:** Autoimmunity, Immunology, Autoimmune diseases, Innate immunity, Lupus

## Abstract

The endosomal Toll-like receptor 7 (TLR7) is a major driver of murine and human systemic lupus erythematosus (SLE). The role of TLR7 in lupus pathogenesis is enhanced when the regulatory role of TLR9 is absent. TLR7 signaling in plasmacytoid DCs (pDC) is generally thought to be a major driver of the IFN response and disease pathology; however, the cell types in which TLR7 acts to mediate disease have not been distinguished. To address this, we selectively deleted TLR7 in either CD11c^+^ cells or CD19^+^ cells; using a TLR7-floxed allele, we created on the lupus-prone MRL/lpr background, along with a BM chimera strategy. Unexpectedly, TLR7 deficiency in CD11c^+^ cells had no impact on disease, while TLR7 deficiency in CD19^+^ B cells yielded mild suppression of proteinuria and a trend toward reduced glomerular disease. However, in TLR9-deficient MRL/lpr mice with accelerated SLE, B cell–specific TLR7 deficiency greatly improved disease. These results support revision of the mechanism by which TLR7 drives lupus and highlight a *cis* regulatory interaction between the protective TLR9 and the pathogenic TLR7 within the B cell compartment. They suggest B cell–directed, dual TLR7 antagonism/TLR9 agonism or dual TLR7/9 antagonism as a potential future therapeutic strategy to treat SLE.

## Introduction

Systemic lupus erythematosus (SLE) is an autoimmune disease characterized by immune cell activation and infiltration into multiple organ systems, causing extensive tissue damage (1, 2). Although patients vary in their clinical presentations, the unifying molecular hallmark of SLE is autoantibody production against DNA- and RNA-containing targets, driven by endosomal Toll-like receptors (TLRs) TLR7 and TLR9 (3).

TLR7 is activated by single-stranded RNA (ssRNA), while TLR9 is activated by CpG-rich DNA, and both have been implicated in resisting viral infection (4–6). In the context of SLE, the prevailing hypothesis is that TLRs are aberrantly activated by self-nucleic acids, leading to an initial break in tolerance and further perpetuation of the autoimmune feed-forward loop (3, 7, 8). This hypothesis was first supported by in vitro evidence demonstrating activation of B cells and DCs via signals from immune complexes containing self nucleic acids (9–12).

Overexpression and gene duplication studies in mice have also supported the notion that TLR7 is pathogenic in SLE (13–15). Human studies have implicated the escape of *TLR7* from X-inactivation in immune cells as a potential factor contributing to the greater female preponderance of the disease (16). Other human studies — including a recently published study that highlighted a *TLR7* gain-of-function variant found in humans (17–20) — have shown associations between TLR7 polymorphisms and SLE.

Despite the pathogenic role of TLR7, and despite that TLR7 and TLR9 are highly homologous and are thought to signal in similar ways, TLR9 plays a dominant protective role, while TLR7 is pathogenic in lupus (21–31). Furthermore, these 2 receptors have a regulatory interaction, such that disease exacerbation via loss of TLR9 can be rescued by deleting TLR7 at the same time (23, 28).

Thus, a central question is how TLR7 and TLR9 act differently to modulate SLE. One possibility is that, in contrast to current models, these receptors use distinct signaling pathways such that TLR9 engages a regulatory circuit that TLR7 does not. Another, not mutually exclusive, possibility is that the different effects of TLR7 and TLR9 arise from cell-specific expression and activation (32–40). We began to address these possibilities regarding TLR9 in 2 recent studies. Using TLR9 mutants, we discovered a ligand-dependent, MyD88-independent regulatory role for TLR9. Separately, Tilstra et al. showed that TLR9 mediates protection from SLE predominantly via expression in the B cell compartment (25, 41).

This latter result does not dictate that TLR7 has a similar cell-specific effect. Indeed, the disease-promoting effects of TLR7 could also be mediated in different cell types or multiple cell types; for example, TLR7 may drive disease via plasmacytoid DCs (pDCs) and/or conventional DCs (cDCs), while TLR9 regulates disease in B cells. In fact, TLR7 is expressed in multiple immune cell subsets, including B cells, cDCs, pDCs, macrophages, and monocytes (40, 42), and pDCs express very high levels of TLR7. A current hypothesis is that TLR7 in pDCs drives SLE. pDCs secrete large amounts of type I IFN, which may underlie the type I IFN signature found in some patients with SLE (2, 10, 43–45). TLR7 overexpression resulted in the pathogenic expansion of DCs, implicating them in the promotion of nephritis (13, 46).

Nonetheless, a role in pathogenesis for TLR7 in B cells also seems likely. B cell–specific deletion of MyD88 greatly ameliorated disease (47); presumably, TLR7 is an important upstream receptor activating MyD88. Additionally, mice with B cell–specific Wiskott-Aldrich Syndrome protein (WASp) deficiency, which causes an autoimmune syndrome, had ameliorated disease when TLR7 was eliminated (30, 48, 49). Since the genetic driver of this model is in B cells, it is logical that the role of TLR7 in this case should also be in B cells; however, TLR7 may play more diverse roles in disease potentiation in polygenic models of SLE.

With this background, important steps toward deciphering the TLR7/9 paradox and toward gaining a greater understanding of how TLR7 promotes disease in lupus involve understanding TLR7’s roles in different cell types in a single model. To this end, we created a floxed TLR7 allele directly on the lupus-prone MRL/lpr genetic background and used tissue-specific Cre-mediated deletion, along with BM chimera approaches, to define the CD11c^+^ cell– and B cell–specific roles of TLR7.

## Results

### TLR7 is expressed in B cells, myeloid cells, and DC lineages of MRL/lpr mice.

**Figure 1:**
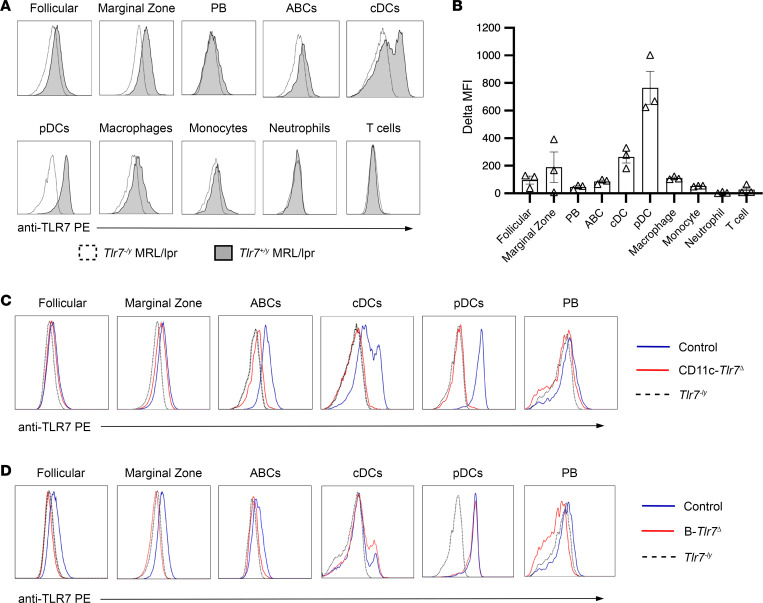
TLR7 is expressed in B cells, DCs, and myeloid cells and is deleted in targeted subsets using Cre-lox approaches. (**A** and **B**) Representative histograms and quantification in the change in anti-TLR7 PE MFI between WT and *Tlr7*^–/y^ for each of the defined populations in 6-week-old male, prediseased MRL/lpr mice. (**C**) Representative histograms showing TLR7 expression in a littermate control (*Tlr7*^fl/y^) mouse (blue line), a CD11c-*Tlr7*^Δ^ (CD11c-Cre^+/–^ *Tlr7*^fl/y^) mouse (red line), and a *Tlr7*^–/y^ mouse (black dotted line) in relevant cell subsets. (**D**) Representative histograms showing TLR7 expression in a littermate control (*Tlr7*^fl/y^) (blue line), a B-*Tlr7*^Δ^ (CD19-Cre^+/–^ *Tlr7*^fl/y^) mouse (red line), and a *Tlr7*^–/y^ mouse (black dotted line) in relevant cell subsets.

TLR7 mRNA is expressed in marginal zone B cells, follicular B cells, some cDC subsets, pDCs, macrophages, and monocytes, with little expression in plasmablasts (40, 42). To extend these data to the protein level, and to confirm that this expression pattern extends to autoimmune-prone MRL/lpr mice, we performed intracellular flow cytometry in prediseased, 8-week-old MRL/lpr mice and calculated the ∆MFI between TLR7WT MRL/lpr and TLR7KO MRL/lpr in each cell subset (Figure 1, A and B). We confirmed expression of TLR7 in marginal zone B cells, CD11c^+^CD11b^+^ B cells (age-associated B cells [ABCs]), cDCs, pDCs, and macrophages. Of these TLR7-expressing subsets, pDCs had the highest expression, with a 4-fold higher MFI compared with marginal zone B cells. There was substantially less expression in follicular B cells, plasmablasts, and monocytes, all of which had expression barely above background levels (Figure 1, A and B). As expected, T cells and neutrophils did not express TLR7 (Figure 1, A and B).

### Selective deletion of TLR7 in CD11c^+^ cells.

Since TLR7 was most highly expressed in pDCs and DCs, we first chose to assess the role of TLR7 in CD11c^+^ populations; the CD11-Cre bacterial artificial chromosome–transgenic (BAC-transgenic) allele is the best available tool to target these 2 populations (50). In addition to cDC and pDC, CD11c-Cre deletes genes in a subset of plasmablasts as well as in ABCs, which express CD11c (51–53). We generated CD11c-Cre^+/–^ *Tlr7*^fl/fl^ (female) and CD11c-Cre^+/–^ *Tlr7*^fl/y^ (male) mice (henceforth referred to as CD11c*-Tlr7*^Δ^) by backcrossing our floxed mice to CD11c-Cre^+/–^ MRL/lpr mice. We performed intracellular flow cytometry on splenocytes from prediseased, 8-week-old MRL/lpr mice, which demonstrated near complete deletion of TLR7 in cDCs and pDCs (with only 4% and 3% of cells retaining TLR7 expression, respectively). We also observed a reduction of TLR7 in the ABCs (although baseline expression of TLR7 in the ABCs was low, as expected in younger mice). TLR7 expression in the CD11c^–^ B cell population was minimally affected. Plasmablasts express little if any TLR7, and its expression was unaffected in Cre^+^ mice (Figure 1C).

### CD11c^+^ cell–specific TLR7 does not affect clinical parameters of SLE pathogenesis in MRL/lpr mice.

**Figure 2:**
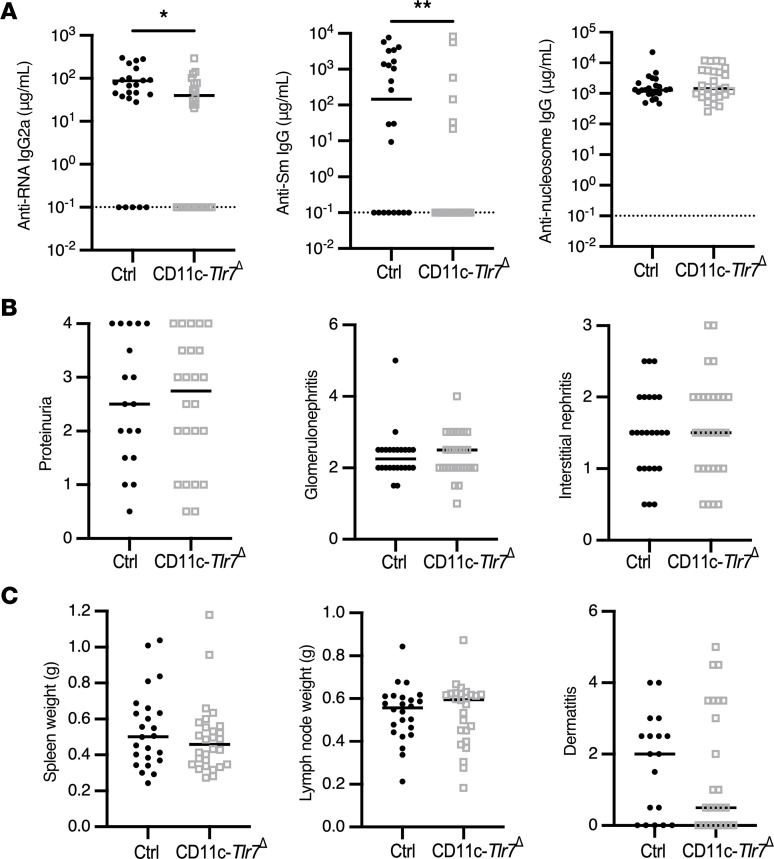
CD11c^+^ cell–specific TLR7 deficiency mildly suppresses the anti-RNA–associated autoantibody response but does not affect clinical parameters of SLE. Control (*Tlr7*^fl/fl^ and *Tlr7*^fl/y^) and CD11c-*Tlr7*^Δ^ (CD11c-Cre^+/–^ *Tlr7*^fl/fl^ and CD11c-Cre^+/–^ *Tlr7*^fl/y^) mice were aged until 19 weeks (female) or 22 weeks (male). (**A**) Serum concentrations of anti–RNA IgG2a, anti–Sm IgG, and anti–nucleosome IgG in control and CD11c-*Tlr7*^Δ^ mice (*n* = 24 and *n* = 28, respectively). (**B** and **C**) Evaluation of phenotypic disease markers in control and CD11c-*Tlr7*^Δ^ mice including proteinuria (*n* = 18 and *n* = 24), glomerulonephritis, and interstitial/perivascular infiltrates (*n* = 24 and *n* = 28) (**B**) and spleen weight, lymph node weight (*n* = 24 and *n* = 28), and dermatitis (*n* = 18 and *n* = 24) (**C**). Scatterplots display data from individual mice, with black lines showing median values and dotted lines indicating the lower limit of detection. *P < 0.05, **P < 0.01 by 2-tailed Mann-Whitney *U* test.

Because female MRL/lpr mice have accelerated disease (54), we aged CD11c*-Tlr7*^Δ^ female mice for 19 weeks and their male littermates for 22 weeks and then evaluated disease, immune activation, and serum autoantibodies. In this experimental cohort of aged mice, deletion was still specific and complete in the DC subsets, but there was residual TLR7 expression in the ABCs, indicating some level of Cre escape in this population after disease onset (Supplemental Figure 1; supplemental material available online with this article; https://doi.org/10.1172/jci.insight.172219DS1). TLR7-driven anti-RNA and anti-Sm autoantibodies were reduced in sera of CD11c*-Tlr7*^Δ^ mice, but TLR9-driven anti-nucleosome autoantibodies were unaffected (Figure 2A and Supplemental Table 1). Since the CD11c-Cre targets ABCs (Figure 1C), which are TLR7 driven and enriched for autoantibody, the selective loss of RNA-associated autoantibodies in a subset of Cre^+^ mice is possibly due to the targeting of TLR7 in these B cells (52, 55). CD11c*-Tlr7*^Δ^ mice exhibited no improvement in any clinical disease parameters — including spleen and lymph node weight, proteinuria, dermatitis, glomerulonephritis, or interstitial nephritis — compared with Cre^–^ controls (Figure 2, B and C). Within the spleen, there were no alterations other than an increase in the proportion of total CD4^+^ T cells in CD11c*-Tlr7*^Δ^ mice (Supplemental Table 2). Analysis of mice separated by sex also revealed no differences in any disease parameters (Supplemental Figure 2 and Supplemental Table 3).

### Selective deletion of TLR7 in CD19^+^ cells.

To examine the effect of TLR7 in B cells, we generated CD19-Cre^+/–^ *Tlr7*^fl/fl^ (female) and CD19-Cre^+/–^ *Tlr7*^fl/y^ (male) MRL/lpr mice (henceforth referred to as B*-Tlr7*^Δ^). To evaluate deletion at the protein level, we performed intracellular flow cytometry in prediseased, 8-week-old B*-Tlr7*^Δ^ mice. Deletion reached 89% in marginal zone B cells with no targeting observed in pDCs or cDCs (Figure 1D). The already low level of TLR7 expression in plasmablasts was unaffected (Figure 1D), consistent with our prior study showing a similar lack of TLR9 targeting in plasmablasts; this is unsurprising, given heterogeneity of CD19 expression on these cells (25, 56). However, in our aged cohort of experimental mice, there was evidence of residual TLR7 expression in the B cells — particularly in marginal zone B cells and ABCs — of some mice, consistent with the notion of Cre escape (Supplemental Figure 1B). Importantly, however, there were no off-target effects on the DC compartments in these aged mice (Supplemental Figure 1).

Because of the residual TLR7 expression observed at the protein level, we performed quantitative PCR (qPCR) on sorted cell subpopulations in 10-week-old mice to assess the extent of *Tlr7* deletion. Deletion in B cells was incomplete, with 79% deletion in females and 80% deletion in males of *Tlr7* alleles in all nonplasmablast B cells (Table 1). Prior data suggest that the CD19-Cre system is typically 95%–97% effective (25). This disparity could be explained if B cells retaining *Tlr7* alleles arose from selective expansion of a small pool of B cells with an undeleted allele (i.e., the 3%–5% normal inefficiency of CD19-Cre), driven by TLR7-mediated activation and selective pressure, contributing to disease phenotypes.

### Deletion of TLR7 in B cells via CD19-Cre improves proteinuria, reduces anti-RNA autoantibodies, and eliminates anti-Sm autoantibodies.

**Figure 3:**
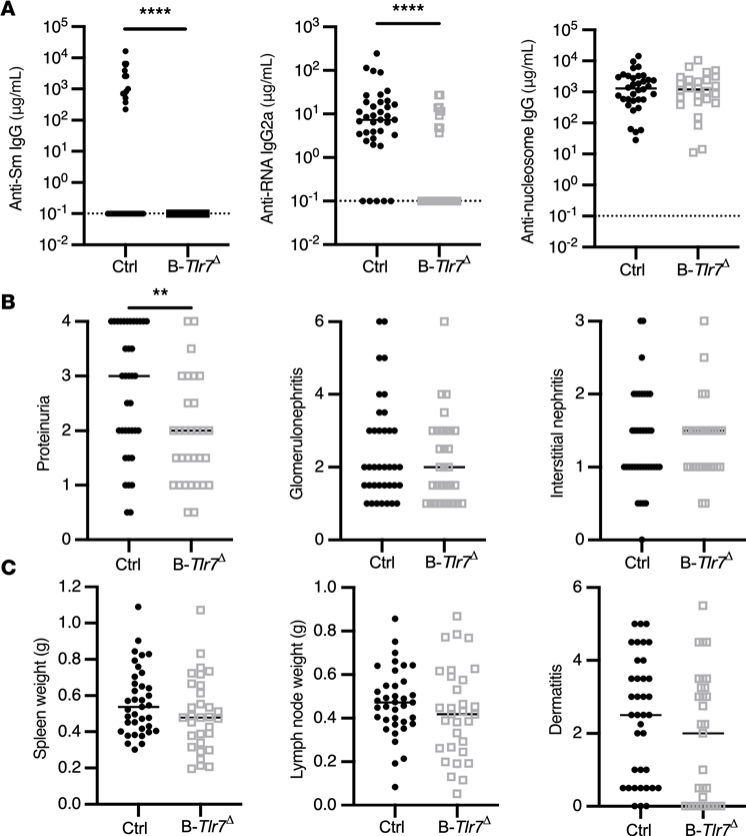
B cell–intrinsic TLR7 deficiency improves proteinuria and suppresses the anti-RNA–associated autoantibody responses. Control (*Tlr7*^fl/fl^ and *Tlr7*^fl/y^) and B-*Tlr7*^Δ^ (CD19-Cre^+/–^ *Tlr7*^fl/fl^ and CD19-Cre^+/–^ *Tlr7*^fl/y^) mice were aged until 19 weeks (female) or 22 weeks (male). (**A**) Serum concentrations of anti–RNA IgG2a, anti–Sm IgG, and anti–nucleosome IgG in control and B-*Tlr7*^Δ^ mice (*n* = 37 and *n* = 31, respectively). (**B** and **C**) Evaluation of phenotypic disease markers in control and B-*Tlr7*^Δ^ mice including proteinuria, glomerulonephritis, and interstitial and perivascular infiltrates (**B**) and spleen and lymph node weight and dermatitis (*n* = 37 control and *n* = 31 B-*Tlr7*^Δ^) (**C**). Scatterplots display data from individual mice, with black lines showing median values and dotted lines indicating the lower limit of detection. **P < 0.01, ****P < 0.0001 by 2-tailed Mann-Whitney *U* test.

To study the role of B cell–intrinsic TLR7 on immune activation and lupus-like disease, we aged B*-Tlr7*^Δ^ mice as we did for CD11c*-Tlr7*^Δ^ mice. As expected, there was a significant reduction of anti-RNA and a complete elimination of anti-Sm autoantibodies in these mice, with no change in anti-nucleosome autoantibodies (Figure 3A and Supplemental Table 1). B*-Tlr7*^Δ^ mice had reduced proteinuria but no significant decrease in either glomerulonephritis or interstitial nephritis (Figure 3B and Supplemental Table 3). There was no alteration in dermatitis, spleen or lymph node weight, or immune cell proportions in the spleens of B*-Tlr7*^Δ^ mice compared with controls (Figure 3C and Supplemental Table 2). There was a significant reduction of proteinuria and lymph node weight in males with a trend toward reduced proteinuria in females (*P* = 0.1); no other disease parameters were affected (Supplemental Figure 3 and Supplemental Table 3). We previously determined that the CD19-Cre allele alone (CD19-Cre^+/–^ MRL/lpr mice) has no effect on disease; thus, it is not responsible for the reduction in proteinuria or the reduction in autoantibodies observed (25).

### Deletion of TLR7 in B cells via a mixed BM chimera mildly suppresses lupus nephritis and improves B cell lymphopenia in female mice.

**Figure 4:**
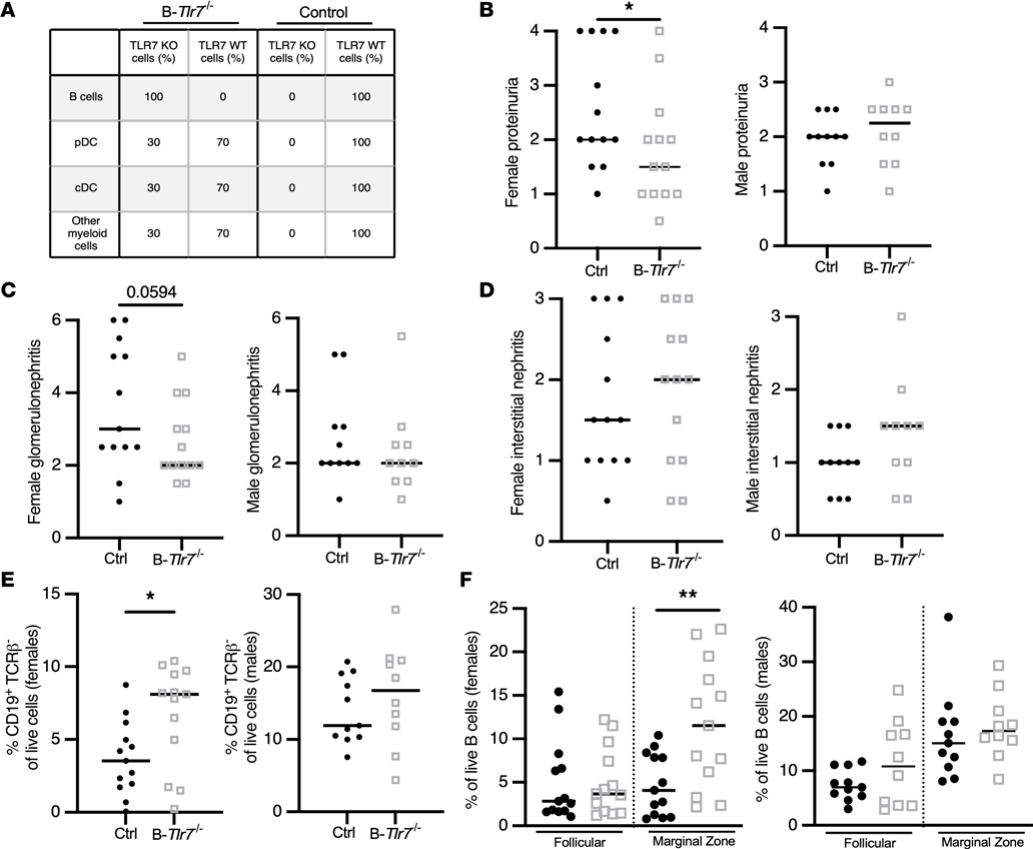
B cell–intrinsic TLR7 deficiency in mixed BM chimeras suppresses features of SLE in female mice. Mixed radiation BM chimeric mice were generated and were then analyzed 25 weeks after irradiation. (**A**) Theoretical reconstitution in the mixed BM chimeras indicating the expected genotype and composition of each listed cellular compartment. (**B**–**D**) Evaluation of clinical SLE parameters in control versus B-*Tlr7*^–/–^ female (*n* = 13 and *n* = 13, respectively) and male (*n* = 11 and *n* = 10, respectively) mice, including proteinuria (**B**), glomerulonephritis (**C**), and interstitial nephritis (**D**). (**E** and **F**) Total B cell (**E**) and follicular and marginal zone B cell (**F**) frequencies in male and female B-*Tlr7*^–/–^ mice compared with controls. Scatterplots display data from individual mice, with black lines showing median values. *P < 0.05, **P < 0.01 by 1-tailed Mann-Whitney *U* test.

The incomplete deletion of TLR7 in B cells may have contributed to the limited phenotype we observed in B*-Tlr7*^Δ^ mice; previously, we observed that — when targeted by the CD19-Cre allele — other disease protective loci, such as *Myd88* (47) and *MhcII* (57), were also inefficiently deleted, suggesting expansion of escaped pathogenic cells. Therefore, to better enforce B cell–specific TLR7 deletion, we generated mixed BM chimeras with a mixture of 70% *Jh* MRL/lpr and 30% *Tlr7*^–/–^ MRL/lpr BM such that all B cells were TLR7 deficient while other lineages were 70% TLR7 sufficient (referred to as B*-Tlr7*^–/–^ mice for simplicity, noting that females are B*-Tlr7*^–/–^ and males are B*-Tlr7*^–/y^; Figure 4A). B*-Tlr7*^–/–^ mice aged for 25 weeks after chimerization showed a trend toward reduced glomerulonephritis (*P* = 0.066) (Supplemental Figure 4 and Supplemental Table 3). This was driven by the female mice, which exhibited a significant reduction in proteinuria and a trend toward reduced glomerulonephritis (*P* = 0.059) (Figure 4, B and C, and Supplemental Table 3); male experimental and control mice were not different in these 2 parameters. Neither sex demonstrated a difference in interstitial nephritis (Figure 4D and Supplemental Table 3). The females in the cohort also had less marked B cell lymphopenia, a common feature of murine and human lupus (Figure 4E), driven by an increase in proportion of marginal zone B cells (Figure 4F).

### B cell–specific TLR7 drives severe lupus nephritis in TLR9-deficient MRL/lpr mice.

Although B cell–specific deletion of TLR7 using both strategies demonstrated some expected effects of reduction in disease markers and autoantibodies, these effects were rather mild. Notably, as we previously published, even global deletion of TLR7 resulted in relatively minor disease reduction in MRL/lpr mice (22). However, in subsequent work, we found that the effects of TLR7 global deletion were stronger and more evident in the context of global TLR9 deletion; such TLR9 deletion on its own exacerbates disease (23). Thus, we hypothesized that the global absence of TLR9 may impact the way B cell–expressed TLR7 affects lupus. To test this genetically, we crossed B*-Tlr7*^Δ^ mice to global *Tlr9*^–/–^ MRL/lpr mice (referred to as B*-Tlr7*^Δ^ *TLR9*^–/–^ mice) and aged cohorts for 16 weeks (females) and 19 weeks (males). These shorter aging durations were mandated by the accelerated disease seen in *Tlr9*^–/–^ MRL/lpr mice (22).

**Figure 5:**
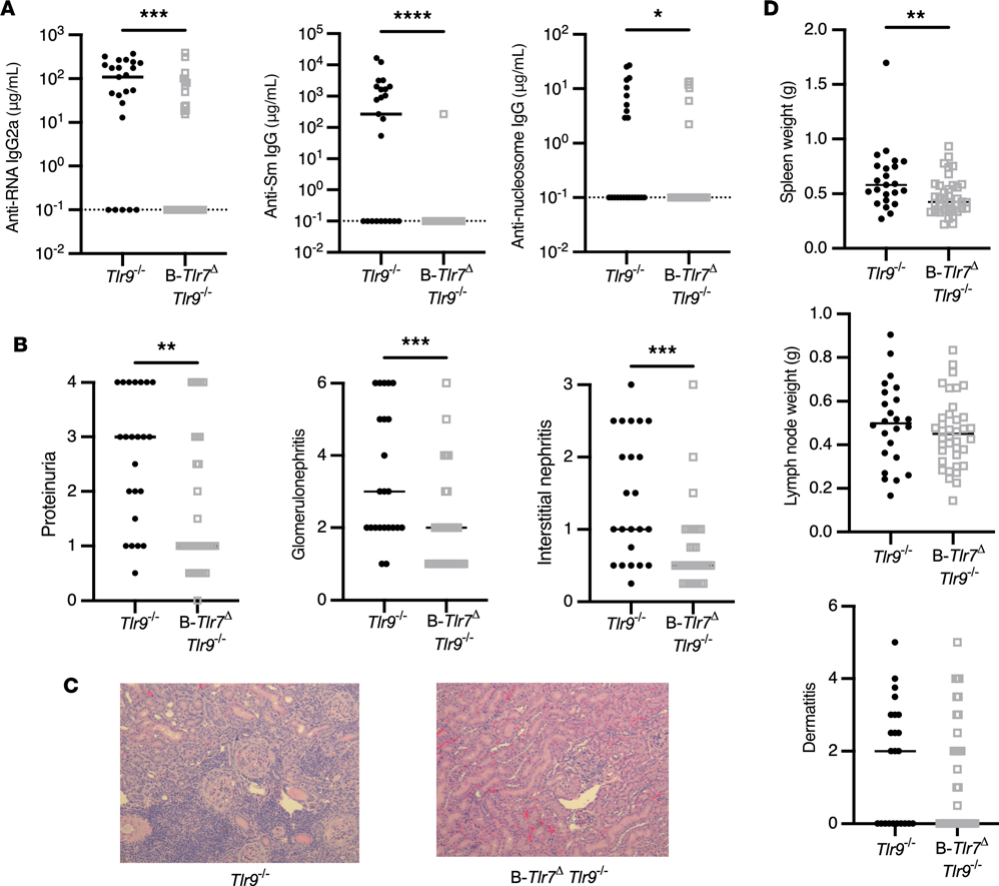
B cell–intrinsic TLR7 drives severe renal disease, splenomegaly, and the anti-RNA–associated autoantibody responses in TLR9-deficient mice. Control (*Tlr7*^fl/fl^ *Tlr9*^–/–^ and *Tlr7*^fl/y^ *TLR9*^–/–^) and B-*Tlr7*^Δ^ *Tlr9*^–/–^ (CD19-Cre^+/–^ *Tlr7*^fl/fl^ *Tlr9*^–/–^ and CD19-Cre^+/–^ *Tlr7*^fl/y^ *Tlr9*^–/–^) mice were aged until 16 weeks (female) or 19 weeks (male). (**A**) Serum concentrations of anti–RNA IgG2a, anti–Sm IgG, and anti–nucleosome IgG in control and B-*Tlr7*^Δ^ *Tlr9*^–/–^ (*n* = 23 and *n* = 37, respectively). (**B**) Evaluation of renal disease including proteinuria, glomerulonephritis, and interstitial and perivascular infiltrates in control versus B-*Tlr7*^Δ^ *Tlr9*^–/–^ mice (*n* = 23 and *n* = 37, respectively). (**C**) Representative images of H&E-stained kidney sections for indicated genotypes. Original magnification, 200×. (**D**) Quantification of spleen weight, lymph node weight, and dermatitis in control versus B-*Tlr7*^Δ^ *Tlr9*^–/–^ mice (*n* = 23 and *n* = 37, respectively). Scatterplots display data from individual mice, with black lines showing median values and dotted lines indicating the lower limit of detection. *P < 0.05, **P < 0.01, ***P < 0.001, ****P < 0.0001 by 1-tailed Mann-Whitney *U* test.

Aged B*-Tlr7*^Δ^ *Tlr9*^–/–^ mice had a similar pattern of TLR7 deletion when compared with B*-Tlr7*^Δ^ mice, again indicating that escape occurs in a subset of B cells (Supplemental Figure 1). Like B*-Tlr7*^Δ^ mice, the B*-Tlr7*^Δ^ *Tlr9*^–/–^ mice also exhibited significantly reduced anti-RNA and anti-Sm autoantibody titers. As expected for global *Tlr9*^–/–^ mice, both experimental and control animals lacked anti-nucleosome autoantibodies (Figure 5A and Supplemental Table 1). Despite escape from complete *TLR7* deletion in the B cell compartment (Supplemental Figure 1C), B*-Tlr7*^Δ^ *Tlr9*^–/–^ mice exhibited a significant decrease in proteinuria (Figure 5B and Supplemental Table 3). Critically, histologic analysis revealed drastically reduced glomerulonephritis and interstitial nephritis in B*-Tlr7*^Δ^ *TLR9*^–/–^ mice compared with *Tlr9*^–/–^ controls (Figure 5, B and C, and Supplemental Table 3). Evaluation of clinical data by sex showed the improvement in proteinuria was driven by females; however, improvement in glomerulonephritis and interstitial nephritis was seen in both sexes (Supplemental Figure 5 and Supplemental Table 3). Thus, TLR7 deficiency exclusively in B cells is sufficient to ameliorate the exacerbated renal disease observed in MRL/lpr mice globally deficient for TLR9.

### B cell–specific TLR7 drives some but not all lymphoid abnormalities in TLR9-deficient mice.

**Figure 6:**
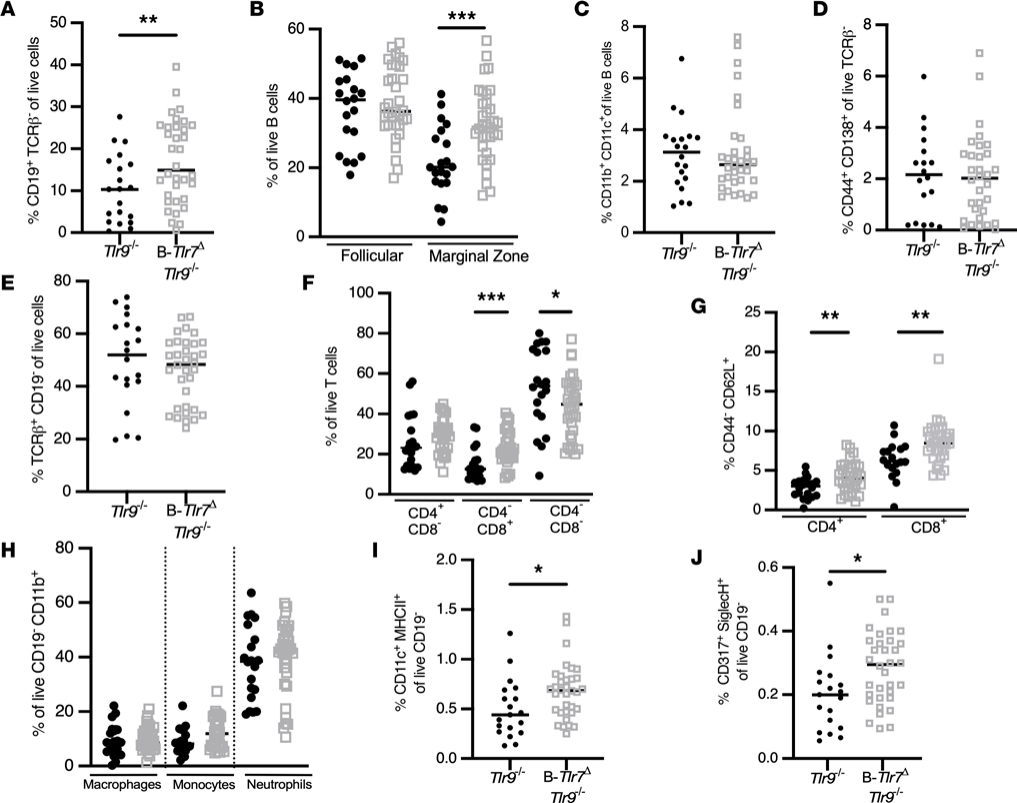
B cell–intrinsic TLR7 drives naive T cell activation, double-negative T cell expansion, and B cell lymphopenia in TLR9-deficient mice. Flow cytometric analysis was performed on spleens from control (*Tlr7*^fl/fl^ *Tlr9*^–/–^ and *Tlr7*^fl/y^ *Tlr9*^–/–^, solid black circles) and B-*Tlr7*^Δ^ *Tlr9*^–/–^ (CD19-Cre^+/–^ *Tlr7*^fl/fl^ *Tlr9*^–/–^ and CD19-Cre^+/–^ *Tlr7*^fl/y^ *Tlr9*^–/–^, open gray squares) mice. (**A**–**D**) Analysis of the B cell compartment in control versus B-*Tlr7*^Δ^ *Tlr9*^–/–^, including proportions of B cells as percent of total live cells (**A**), follicular and marginal zone B cells as a percent of total B cells (**B**), CD11b^+^CD11c^+^ ABCs as a percent of total B cells (**C**), and plasmablasts as a percent of total TCRβ^–^ cells (**D**). (**E**–**G**) Analysis of the T cell compartment in control versus B-*Tlr7*^Δ^ *TLR9*^–/–^, including proportions of T cells as a percent of total live cells (**E**); CD4^+^, CD8^+^, and double-negative (CD4^–^CD8^–^) T cells as a percent of total T cells (**F**); and percent naive CD4^+^ and naive CD8^+^ T cells as a percent of CD4^+^ and CD8^+^ T cells, respectively (**G**). (**H**–**J**) Analysis of selected myeloid populations, including proportions of macrophages, monocytes, and neutrophils as a percent of total CD19^–^CD11b^+^ cells (**H**); cDCs as a percent of total CD19^–^ cells (**I**); and pDCs as a percent of total CD19^–^ cells (**J**). Scatterplots display data from individual mice, with black lines showing median values. *P < 0.05, **P < 0.01, ***P < 0.001, by 1-tailed Mann-Whitney *U* test.

Overall, B*-Tlr7*^Δ^ *Tlr9*^–/–^ mice exhibited significantly reduced spleen weight but no reduction in lymphadenopathy or dermatitis (Figure 5D and Supplemental Table 3). Within the splenic B cell compartment, there was an improvement in B cell lymphopenia, with an increased frequency of marginal zone B cells (Figure 6, A and B). There was no change in the proportion of ABCs or plasmablasts (Figure 6, C and D). The overall splenic T cell proportion was unchanged, but there was an increase in proportion of total CD8^+^ T cells (Figure 6, E and F). Most importantly, in B*-Tlr7*^Δ^ *Tlr9*^–/–^ mice, there was an increase in the proportion of naive CD4^+^ and naive CD8^+^ T cells (Figure 6, F and G). B*-Tlr7*^Δ^ *Tlr9*^–/–^ mice also showed an increase in the proportion of pDCs and cDCs, with no change in proportion of macrophages, monocytes, or neutrophils (Figure 6, H–J); all cellular changes are summarized in Supplemental Table 2. The loss of TLR7 in the B cell is sufficient to suppress several of the hallmark cellular manifestations of SLE in *Tlr9*^–/–^ MRL/lpr mice, including T cell activation and B cell lymphopenia.

## Discussion

The ssRNA-sensing receptor TLR7 is implicated as a driver of lupus pathology in both murine models and human disease. TLR7 is expressed in multiple immune cell subsets, including B cells, pDC, cDC, macrophages, and monocytes (40, 42). However, despite the prevailing view that DC-intrinsic TLR7 drives the manifestations of SLE, whether TLR7 acts in DCs, B cells, or both has not been genetically tested in an unbiased approach. Here we used CD11c^+^ cell– and B cell–specific KOs of TLR7 using both the Cre-lox system and a mixed BM chimera strategy to directly test cell-specific functions of TLR7 in a mouse model of lupus that initially highlighted the role of TLR7.

While we found no significant lupus-related phenotypes in CD11c*-Tlr7*^Δ^ mice, even though deletion of TLR7 in cDC and pDC was highly effective, eliminating TLR7 expression in B cells had a mild effect on clinical lupus. In contrast, and most interestingly, there was a much more penetrant effect of B cell–specific TLR7 deletion when TLR9 was absent in the mouse. Hence, we conclude that TLR7 promotes lupus by acting in B cells — not pDC or DC — particularly when the regulatory effect of TLR9 is absent.

The lack of effect in DC fits with prior observations that deletion of *MyD88* in CD11c^+^ cells did not impact clinical disease (47) and that deletion of *Tlr9* in CD11c^+^ cells was not protective (25). On the other hand, our studies found no role for TLR7 in DCs, and this seem somewhat in conflict with a few prior studies. There were a DC expansion and some signs of autoimmunity when *Tlr7* was globally overexpressed in multicopy on the B6.*Sle1* background (a lupus-prone strain harboring the *Sle1* susceptibility locus). When *Tlr7* expression from this BAC Tg was eliminated using CD11c-Cre, autoimmune pathology and autoantibodies were reduced (13, 46). Those studies did not test the role of TLR7 in the context of TLR9 deficiency, however. The reduced disease and reduced autoantibodies observed in prior studies may also have been due to deletion of TLR7 in CD11c^+^ B cells (ABC). There may be model-specific differences, particularly as the BAC Tg model starts out from a baseline of TLR7 overexpression. Thus, based on prior studies and the current study, a plausible explanation is that, while TLR7 signaling in cDCs may contribute to disease, it is not required for disease.

It has also been proposed that TLR7 in pDCs may drive clinical manifestations of SLE. In vitro studies in humans show that anti-nuclear antibodies containing immune complexes (ICs) will promote pDC activation and IFN-I secretion in a presumably TLR7-dependent fashion (10, 58, 59), but it is difficult to establish in humans whether this promotes disease in vivo. BXSB male mice, which develop lupus due to the *Yaa* locus on the Y chromosome, had ameliorated disease with early depletion of the pDC population, but whether this was dependent on pDC-intrinsic TLR7 was not tested (60). Mice overexpressing TLR7 from a BAC Tg had ameliorated glomerulonephritis and reduced germinal centers when pDC function was impaired via haploinsufficiency of *Tcf4*, implying a role for pDCs. However, haploinsufficiency of *Tcf4* has since been identified as a key regulator of germinal center B cells and plasma cells (61, 62). In our mice, using CD11c-Cre, TLR7 protein expression was very effectively eliminated, yet there was no demonstrable effect on disease.

The results from this study do define a B cell–specific role for TLR7 in driving lupus pathology. This finding is commensurate with the role of B cell–intrinsic MyD88 in driving SLE (47). Since we also know that TLR9’s negative regulation of lupus pathology requires its expression in B cells (25), this suggests that it may directly interact with TLR7 in some fashion, since TLR7’s role in driving disease is also B cell intrinsic (25, 41, 63). Jackson and colleagues similarly found a B cell–intrinsic role for TLR7 in an autoimmune model based on B cell–specific deletion of WASp. Since the driving genetic defect in this model is in B cells by design, it is perhaps not surprising that TLR7 also would have a B cell–intrinsic effect in these mice (30). Nonetheless, it is encouraging that different models of murine lupus demonstrate concordant results.

Mechanistically, it is possible that B cell–intrinsic TLR7 promotes an ABC-like phenotype to drive SLE. ABCs are known to drive autoimmunity, are enriched for autoantibody specificities, are efficient antigen presenters, and require TLR7 or TLR9 signaling for formation in vitro (51, 55, 64). In our work, though, there was not a decrease in the proportion of CD11b^+^CD11c^+^ ABCs when TLR7 was deleted, seemingly arguing against this concept. However, leaky Cre-mediated deletion of *Tlr7*, particularly among the ABCs, likely still allowed for ABC formation and the selective expansion of ABCs that retained TLR7. If so, this further indicates that TLR7 does play a role by promoting ABC formation. In addition, TLR7 could also affect ABC function, and the deletion of TLR7 that did occur may have been sufficient to blunt ABC function and thereby ameliorate disease.

Overall, there was only modest suppression of SLE clinical parameters in B*-Tlr7*^Δ^ mice. Our prior data (22, 23) consistently demonstrate that global deletion of *Tlr7* resulted in only a modest reduction in renal disease compared with WT controls. Hence, the phenotype seen in the B cell–specific TLR7 KO is quite similar to that seen in the global KO. However, a much more potent effect was evident when *Tlr7* was globally deleted in mice that had accelerated disease due to concurrent *Tlr9* deletion (23). Thus, the striking improvement in multiple disease parameters in our B*-Tlr7*^Δ^ *Tlr9*^–/–^ mice also fits with the phenotype of global deletion, further arguing that TLR7 acting in the B cell accounts for most, if not all, of its overall effects.

The more dramatic effect of TLR7 deletion in the context of TLR9 deficiency can be interpreted in light of our recent study showing that TLR9 has dual B cell–intrinsic roles: (a) a Myd88-independent, regulatory activity that restrains activation and differentiation of ABCs and plasmablasts and (b) MyD88-dependent, disease-promoting activity (41). This, in combination with the present study, suggests that TLR7 signaling in B cells is pathogenic but could be restrained by TLR9’s protective effect under normal circumstances. When TLR7 is deleted, the MyD88-driven proinflammatory function of TLR9 may compensate, thus limiting the apparent protective effect of TLR7 deletion. However, when TLR7 is no longer restrained by the protective signaling of TLR9, TLR7 can drive severe manifestations of SLE. When both TLRs are deleted, then B cells lack any endosomal MyD88-dependent stimulation, leading to the lowest level of disease.

In addition to providing important data on the question of how TLR9 and TLR7 cross-regulate, by showing the B cell–intrinsic role of TLR7, the present study has practical implications for developing new SLE therapies; this is particularly important given the association between human *TLR7* polymorphisms and gain-of-function variants and SLE. For instance, simultaneous TLR7/9 antagonism and TLR7 antagonism plus TLR9 agonism targeted to B cells — or, specifically, ABCs — represent potential treatment strategies that should be further explored and, if technically achievable, may provide more specific disease amelioration without global immune suppression.

## Methods

### Generation of a conditional TLR7 allele.

A conditional allele of *Tlr7* was generated using CRISPR/Cas9 technology directly in MRL-MpJ-*Fas*^lpr^/J eggs. Briefly, the targeting strategy introduced 2 LoxP sites flanking the coding sequence of TLR7. Fertilized embryos produced by natural mating of MRL/lpr mice were microinjected in the cytoplasm with Cas9 mRNA (100 ng/μL), *Tlr7*-proximal guide and *Tlr7*-distal3 guide (50 ng/μL each), and 2 single-stranded oligonucleotides (*Tlr7*-proximal-HDR and *Tlr7*-distal-HDR, 0.5 μM; Integrated DNA Technologies). Injected zygotes were cultured overnight, and 2 cell embryos were transferred to pseudopregnant CD1.

Cas9 mRNA and the sgRNA were produced as described (65). The target sequences of the sgRNAs are 5′-ATCTGAGACACCTAATTGGAGGG-3′ for the *Tlr7*-proximal guide at chrX:167,309,786-167,309,808 and 5′-TTCATAGTAAGCCCAAAAGGGGG-3′ for the *Tlr7*-distal3 guide at chrX:167,303,821-167,303,843. The genomic locations are from the GRCm38/mm10 assembly.

The sequences of the single-stranded oligonucleotides are 5′-ATCTATGATGATTTGTTGTAGTTTCCCTGCATACTGGGGTTTGTTTGGTTTCAGGATCTGAGACACCTAATTATAACTTCGTATAATGTATGCTATACGAAGTTATGAATTCGGAGGGAGAGACACAGAGTCAGATCAGTCTTCAGACAAAGGGTTTCATAGAGCTGACAAAACATCAAGATTTGAAGGAAGGAA-3′ for the *Tlr7*-proximal-HDR and 5′-TTTATGAACTAGTACCAATATTTTTCAGTATCTATAACTATCATGCATCAATATCAAGTGGTTTTCATAGTAAGCCCAAAGAATTCATAACTTCGTATAGCATACATTATACGAAGTTATAGGGGGTGGAAAGAATATGTATTTAGTGTTATCAGGGAATAGGAAAATAGATGGATGGTAATTGTATATGTTTAC-3′ for the *Tlr7*-distal3-HDR.

The target sites in the potential founders were amplified by PCR, with the region surrounding target *Tlr7*-proximal-HDR detected with primers *Tlr7*-KO-F51 (5′-TGGCACAACACCTTCCTATCT-3′) and *Tlr7*-KO-R51 (5′-GGGATGTGGGTAAGGAATGCT-3′) and the region surrounding the *Tlr7*-distal2 region detected with primers Tlr7-KO-F32 (5′-ATTCCTGCTGAAATATTCTATGTGC-3′) and *Tlr7*-KO-R32 (5′-GGGATTGCCAGTCAGCTAGT-3′) for the *Tlr7*-distal3 region. The PCR product was digestible with EcoRI (site inserted adjacent to the LoxP sites), and the expected sequences were confirmed by Sanger sequencing.

*Tlr7*^fl/fl^ (female) and *Tlr7*^fl/y^ mice (male) were routinely genotyped with the following primers that detect both the WT (250 bp) and floxed alleles (290 bp): *Tlr7*-CR-Fwd 5′-TGCTTCTGCCATTCTGTCATGT-3′ and *Tlr7*-CR-Rev 5′-CCAGATTCCTCTCTCCCCAA-3′.

### Mice.

CD19-Cre and CD11c-Cre mice were backcrossed to MRL-MpJ-*Fas^lpr^*/J as previously described (47). Experimental cohorts on the *Tlr9*^+/+^ background were generated by intercrossing female Cre^+/–^ *Tlr7*^fl/fl^ or male Cre^+/–^ *Tlr7*^fl/y^ mice with male *Tlr7*^fl/y^ or female *Tlr7*^fl/fl^ MRL-MpJ-*Fas^lpr^*/J mice, respectively. Experimental cohorts on the *Tlr9*^–/–^ background were generated by intercrossing Cre^+/–^ *Tlr7*^fl/fl^ or Cre^+/–^ *Tlr7*^fl/y^ mice with *Tlr9*^–/–^ MRL-MpJ-*Fas^lpr^*/J mice until both the *Tlr7*-floxed and *Tlr9*^–/–^ alleles were homozygous. In all cases, littermates without a Cre allele were used as controls, and mice were aged to indicated time points.

For mixed BM chimeras, 7- to 8-week-old B cell–deficient *Jh* (*Igh-J^tm1Dhu^*) MRL-MpJ-*Fas*^lpr^/J mice were irradiated twice with 400 cGy and injected i.v. with a mixture of 7 ***×*** 10^6^ *Jh* MRL-MpJ-*Fas*^lpr^/J together with 3 ***×*** 10^6^ *Tlr7*^–/–^ MRL-MpJ-*Fas*^lpr^/J BM cells. Controls within the same experiment were generated by injecting a mixture of 7 ***×*** 10^6^ *Jh* MRL-MpJ-*Fas*^lpr^/J with 3 ***×*** 10^6^ MRL-MpJ-*Fas*^lpr^/J BM cells. Chimeric mice were analyzed at 24–25 weeks after irradiation.

### Evaluation of clinical disease.

Proteinuria was measured by Albustix strips (Bayer). Kidneys were removed, bisected, formalin fixed, paraffin embedded, and H&E stained. Glomerular and interstitial nephritis were scored by a pathologist in a blinded manner as previously described (25).

Dermatitis was scored based on the extent of dermatitis on the dorsum of the neck and back. The macroscopic surface area was scored from 0 to 5 for an affected area up to 9.1 cm^2^, with up to 1 additional point for the presence of ear (a quarter point each) and muzzle (a half point) dermatitis.

### Flow cytometry.

Spleens were processed via mechanical dissociation, and RBCs were lysed with ammonium-chloride-potassium buffer (in house). Live/dead discrimination was performed using GhostDye Violet 510 or GhostDye UV450 (Tonbo Biosciences). Surface staining was performed in ice-cold PBS (Cytiva) + 0.5% BSA (GeminiBio) + 2.5% HEPES (Corning) + 2.5 mM EDTA (Fisher Bioreagents). Cells were stained in the presence of FcR blocking Ab 2.4G2 (in-house). Intracellular staining was performed using the BD Biosciences Cytofix/Cytoperm kit. Data were collected using an LSRII (BD Biosciences) with FACSDiva (BD Biosciences) and analyzed using FlowJo software (Tree Star Inc.).

### qPCR.

To assess deletion efficiency of *Tlr7* at the genomic level in CD19-Cre mice, genomic DNA was extracted from BD Biosciences FACSAria II–sorted cells for qPCR. The amount of *Tlr7* was normalized to *Tlr9*, and male and female analyses were performed separately since *Tlr7* is located on the X chromosome. qPCR was performed with Agilent Brilliant II SYBR Green qPCR kit on a Roche LightCycler 96.

### Measurement of serum autoantibodies.

Anti-nucleosome, anti-RNA, and anti-Sm concentrations in serum were measured by ELISA as previously described (23, 66). Specific antibodies were detected with alkaline phosphatase–conjugated goat anti–mouse IgG (Southern Biotech, 1030-04) or IgG2a (Southern Biotech, 1080-04). The monoclonal antibodies PL2-3, BWR4, or Y2 (in-house) were used as standards for the anti-nucleosome, anti-RNA, and anti-Sm measurements, respectively.

### Antibodies.

Antibodies used for FACS surface and intracellular staining were as follows: IA/E-BV605 (BioLegend, M5/114.15.2), CD11c-PE/Cy7 (in-house conjugated, N418), SiglecH-Al647 (BioLegend, 551), CD19-BUV-395 (BD Horizon, 1D3), CD44-Al488 (in-house conjugated, 1M7), CD21/35-PerCP/Cy5.5 (BioLegend, 7E9), CD23-biotin (in-house conjugated, B3B4), CD23 PE/Cy7 (BioLegend, B3B4), CD317-Biotin (eBioscience, eBio927), Gr1-PE/Cy7 (BioLegend, RB6-8C5), CD11b-biotin (in-house conjugated, M1/70), CD11b-PacBlue (in-house conjugated, M1/70), F4/80-Al647 (in-house conjugated, BM8), TCRβ-APC/Cy7 (BioLegend, H57-597), CD62L-PE/Cy7 (BioLegend, Mel-14), CD8-Al647 (in-house conjugated, TIB 105), CD4-PE (in-house conjugated, GK1.5), CD4-PacBlue (in-house conjugated, GK1.5), CD138-BV605 (BD Horizon, 281-2), Streptavidin-BUV395 (BD Horizon, 564176), Streptavidin-PE/Cy7 (BD Pharmingen, 557598), and TLR7-PE (BD Pharmingen, A94B10).

### Statistics.

Statistics were calculated in GraphPad Prism by 1- or 2-tailed Mann-Whitney *U* test. *P* < 0.05 was considered significant.

### Study approval.

All work was approved by the University of Pittsburgh IACUC.

### Data availability.

Values for all data points in graphs are reported in the Supporting Data Values file and from the corresponding authors upon request.

## Author contributions

MJS and JST conceived the study. SG, JST, and MJS developed and validated the TLR7-floxed mice. HAC, MK, and JST performed experiments. HAC and JST analyzed data. SB conducted pathologic analysis of the kidney tissue. MJS and HAC acquired funding. JST and MJS provided project administration and supervision. HAC wrote the original draft. HAC, JST, and MJS edited the draft. HAC, MK, SG, SB, JST, and MJS reviewed and approved the draft.

## Supplementary Material

Supplemental data

Supporting data values

## Figures and Tables

**Table 1:**
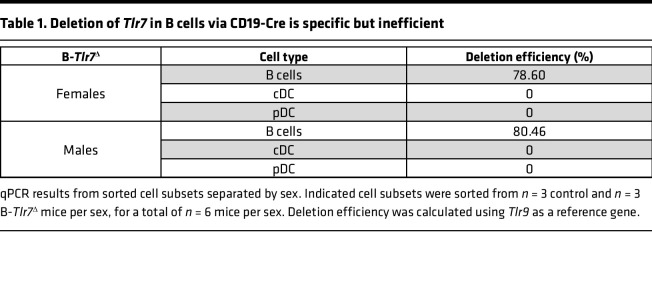
Deletion of *Tlr7* in B cells via CD19-Cre is specific but inefficient

## Version 1 08/22/2023

Electronic publication
