# Supplementary material for: B cell–intrinsic TLR7 expression drives severe lupus in TLR9-deficient mice
Source: JCI Insight. 2023 Aug 22;8(16):e172219. doi: 10.1172/jci.insight.172219 (PMC10543715; doi:10.1172/jci.insight.172219)
Supplement: Supplemental data [file jciinsight-8-172219-s150.pdf]

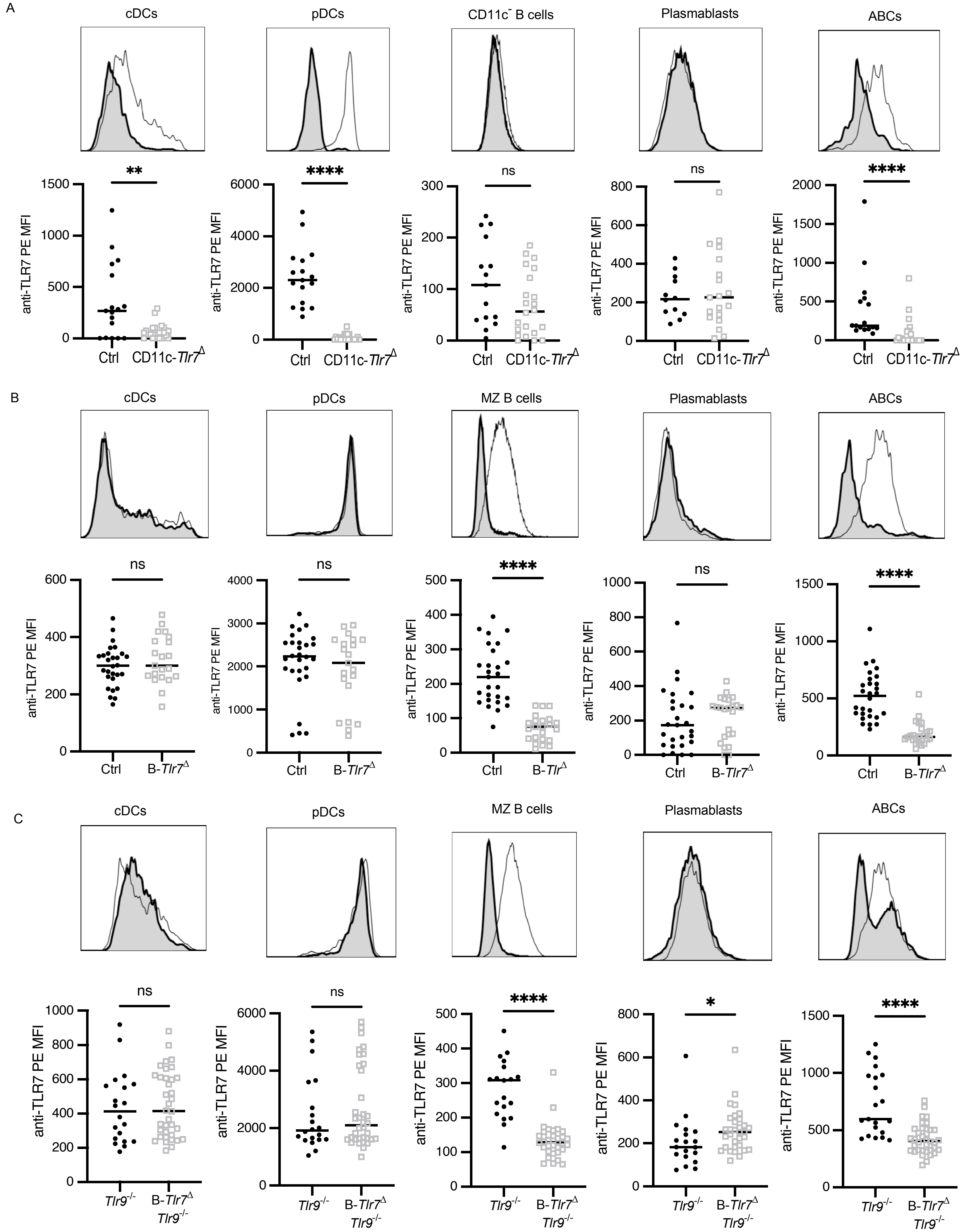

Supplemental Figure 1. Deletion analysis of TLR7 in aged experimental MRL/lpr mice. (A-C) Cre-lox mediated deletion in relevant cell subsets of experimental aged mice via representative histograms and tabulated data of anti-TLR7 PE MFI in the indicated subsets of cells: cDCs ($CD19^-$ $CD11c^+$ $MHCII^+$); pDCs ($CD19^-$ Siglec H^+ $CD317^+$); CD11c- B cells ($TCRb^-$ $CD19^+$ $CD11c^-$); Marginal Zone B cells ($TCRb^-$ $CD19^+$ $CD21/35^+$ $CD23^-$); plasmablasts ($TCRb^-$ $CD44^+$ $CD138^+$); and ABCs ($CD19^+$ $CD11c^+$ $CD11b^+$) **(A)** Control ($Tlr7^{fl/fl}$ and $Tlr7^{fl/y}$) and CD11c-$Tlr7^{\Delta}$ (CD11c-$Cre^{+/-}$ $Tlr7^{fl/fl}$ and CD11c-$Cre^{+/-}$ $Tlr7^{fl/y}$) mice, **(B)** Control ($Tlr7^{fl/fl}$ and $Tlr7^{fl/y}$) and B-$Tlr7^{\Delta}$ (CD19-$Cre^{+/-}$ $Tlr7^{fl/fl}$ and CD19-$Cre^{+/-}$ $Tlr7^{fl/y}$) mice, and **(C)** Control ($Tlr7^{fl/fl}$ $Tlr9^{-/-}$ and $Tlr7^{fl/y}$ $TLR9^{-/-}$) and B-$Tlr7^{\Delta}$ $Tlr9^{-/-}$ (CD19-$Cre^{+/-}$ $Tlr7^{fl/fl}$ $Tlr9^{-/-}$ and CD19-$Cre^{+/-}$ $Tlr7^{fl/y}$ $Tlr9^{-/-}$) mice. For all histograms the light line with open area is the control and the dark line with shaded area is the experimental (Cre x flox) group. Scatter plots display data from individual mice with black lines showing median values. *$P<0.05$, ** $P<0.01$, **** $P<0.0001$ by 2-tailed Mann-Whitney *U* test.

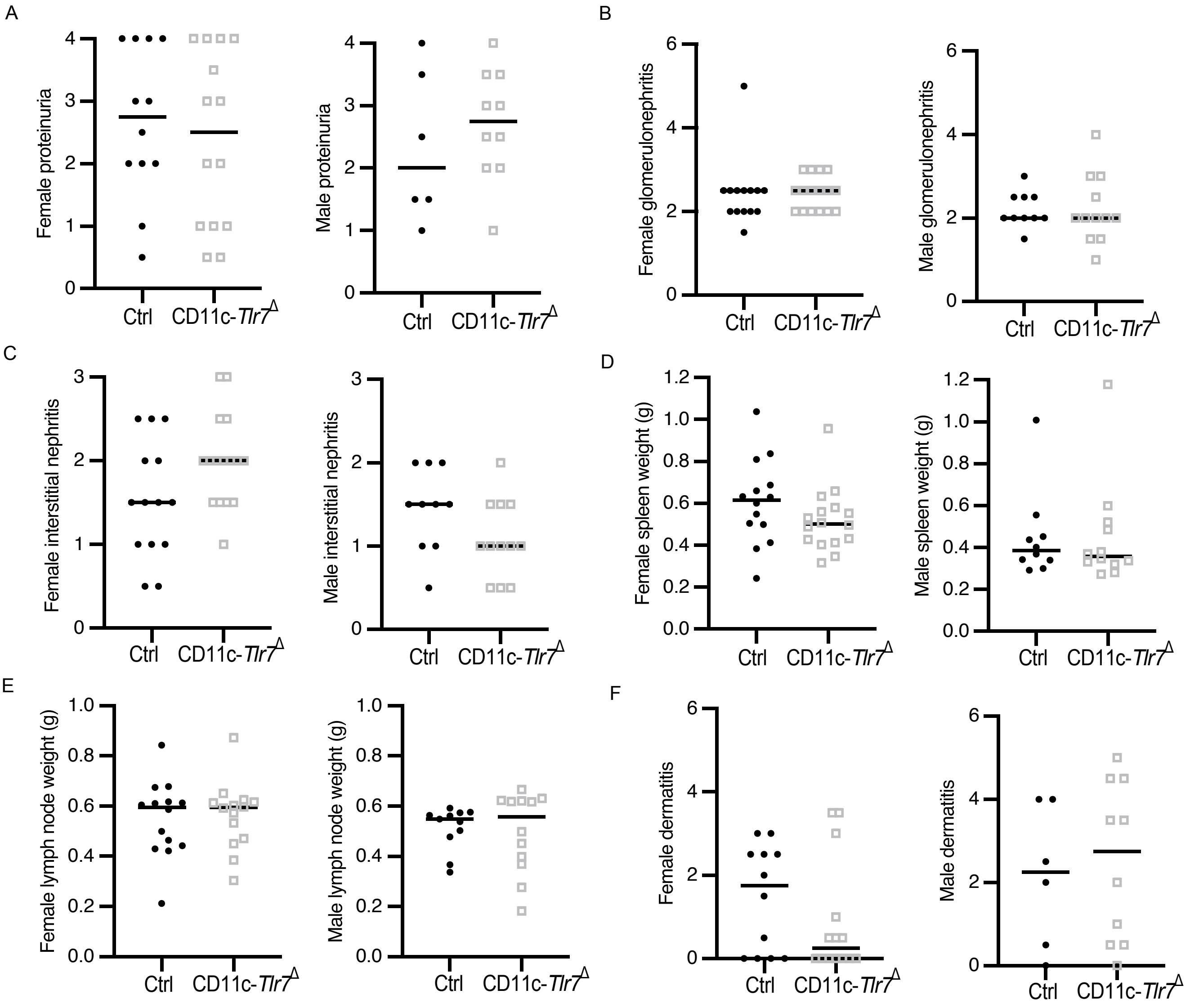

Supplemental Figure 2. Separate analysis of male and female clinical parameters in CD11c-*Tlr7*$^{\Delta}$ mice. Female control (*Tlr7*$^{fl/fl}$) and CD11c-*Tlr7*$^{\Delta}$ (CD11c-Cre$^{+/-}$ *Tlr7*$^{fl/fl}$) mice were aged for 19 weeks. Male control (*Tlr7*$^{fl/y}$) and CD11c-*Tlr7*$^{\Delta}$ (CD11c-Cre$^{+/-}$ *Tlr7*$^{fl/y}$) mice were aged for 22 weeks. **(A-F)** Evaluation of phenotypic disease markers in control and CD11c-*Tlr7*$^{\Delta}$ mice including: **(A)** proteinuria (females, n=12 and n=14; males, n=6 and n=10), **(B)** glomerulonephritis (females n=14 and n=16; males n=10 and n=12), **(C)** interstitial/perivascular infiltrates (females n=14 and n=16; males n=10 and n=12), **(D)** spleen weight (females n=14 and n=16; males n=10 and n=12), **(E)** lymph node weight (females n=14 and n=14; males n=10 and n=12), and **(F)** dermatitis (females n=14 and n=16; males n=6 and n=10). Scatter plots display data from individual mice with black lines showing median values. * P<0.05, ** P<0.01 by 2-tailed Mann-Whitney *U* test.

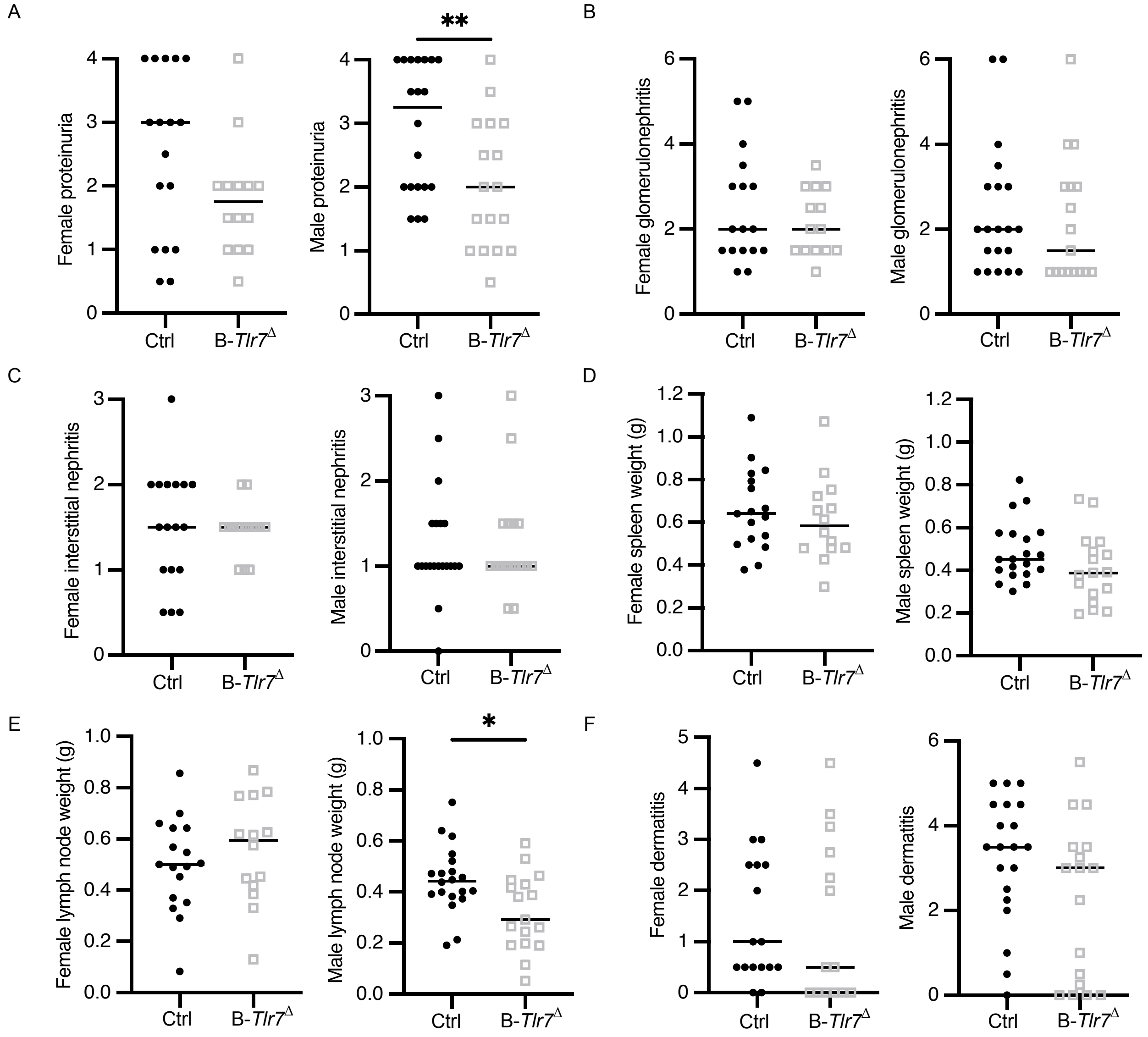

Supplemental Figure 3. Separate analysis of male and female clinical parameters in B-*Tlr7*$^{\Delta}$ mice. Female control (*Tlr7*$^{fl/fl}$) and B-*Tlr7*$^{\Delta}$ (CD19-Cre$^{+/-}$ *Tlr7*$^{fl/fl}$) were aged for 19 weeks. Male control (*Tlr7*$^{fl/y}$) and B-*Tlr7*$^{\Delta}$ (CD19-Cre$^{+/-}$ *Tlr7*$^{fl/y}$) were aged for 22 weeks. **(A-F)** Evaluation of phenotypic disease markers in control and B-*Tlr7*$^{\Delta}$ mice including: **(A)** proteinuria (females, n=17 and n=14; males, n=20 and n=17), **(B)** glomerulonephritis (females n=17 and n=14; males n=20 and n=17), **(C)** interstitial/perivascular infiltrates (females n=17 and n=14; males n=20 and n=17), **(D)** spleen weight (females n=17 and n=14; males n=20 and n=17), **(E)** lymph node weight (females n=17 and n=14; males n=20 and n=17), and **(F)** dermatitis (females n=17 and n=14; males n=20 and n=17). Scatter plots display data from individual mice with black lines showing median values. * P<0.05, ** P<0.01 by 2-tailed Mann-Whitney *U* test.

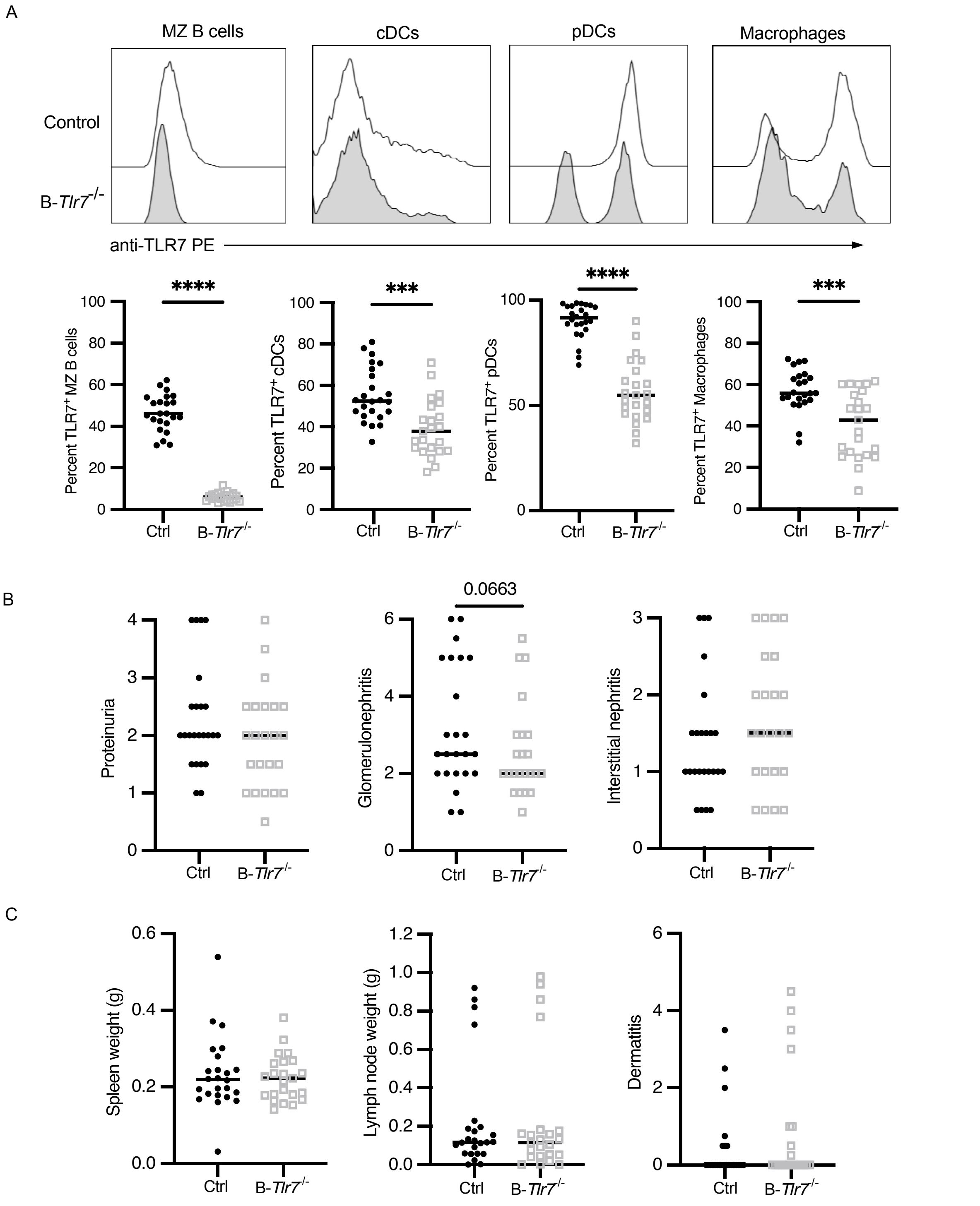

Supplemental Figure 4. TLR7 expression in B-*Tlr7*$^{-/-}$ bone marrow chimeras and combined male and female clinical parameters in the chimeras. (A) Representative histograms showing TLR7 expression by flow cytometry and tabulated data of percent TLR7^{+} cells in control (no fill) and B-*Tlr7*$^{-/-}$ (gray fill) mice in relevant cell subsets: Marginal Zone B cells (TCRb^{-} CD19^{+} CD21/35^{+} CD23^{-}); cDCs (CD19^{-} CD11c^{+} MHCII^{+}); pDCs (CD19^{-} Siglec H^{+} CD317^{+}); and macrophages (CD19^{-} CD11b^{+} LyG/C^{-} F4/80^{+}) **(B)** Combined male and female clinical SLE parameters in control vs B-*Tlr7*$^{-/-}$ mice, n=24 and n=23 respectively. Scatter plots display data from individual mice with black lines showing median values. *** P<0.001, **** P<0.0001 by 1-tailed Mann-Whitney *U* test.

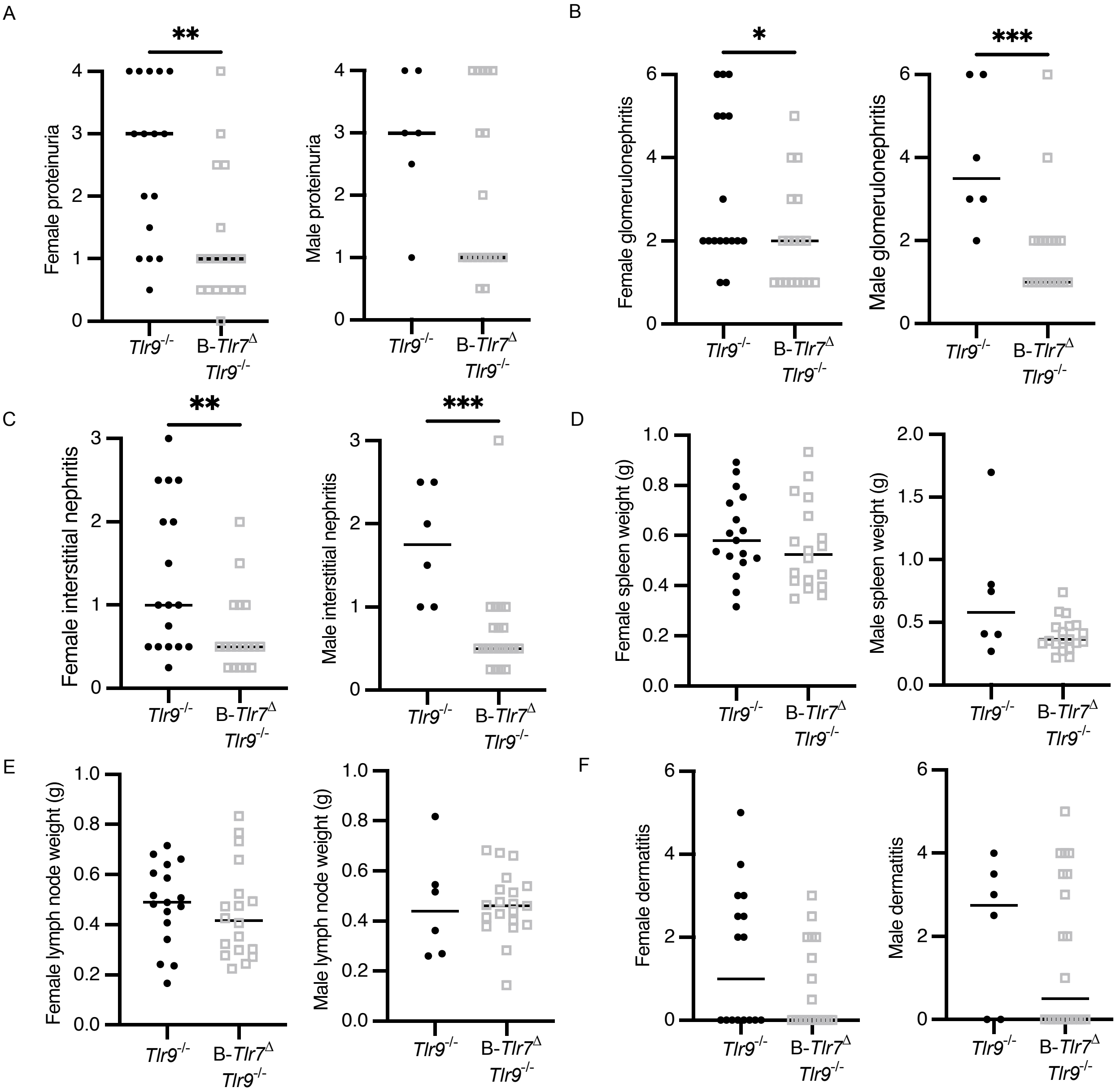

Supplemental Figure 5. Separate analysis of male and female clinical parameters in B-*Tlr7*$^{\Delta}$ *Tlr9*$^{-/-}$ mice. Female control (*Tlr7*$^{fl/fl}$ ^{y}*Tlr9*$^{-/-}$) and B-*Tlr7*$^{\Delta}$ (CD19-Cre$^{+/-}$ *Tlr7*$^{fl/fl}$ *Tlr9*$^{-/-}$) were aged for 16 weeks. Male control (*Tlr7*$^{fl/y}$*Tlr9*$^{-/-}$) and B-*Tlr7*$^{\Delta}$ (CD19-Cre$^{+/-}$ *Tlr7*$^{fl/y}$ *Tlr9*$^{-/-}$) were aged for 19 weeks. **(A-F)** Evaluation of phenotypic disease markers in control and B-*Tlr7*$^{\Delta}$ mice including: **(A)** proteinuria (females, n=17 and n=18; males, n=6 and n=19), **(B)** glomerulonephritis (females n=17 and n=18; males n=6 and n=19), **(C)** interstitial/perivascular infiltrates (females n=17 and n=18; males n=6 and n=19), **(D)** spleen weight (females n=17 and n=18; males n=6 and n=19) **(E)** lymph node weight (females n=17 and n=18; males n=6 and n=19), and **(F)** dermatitis (females n=16 and n=18; males n=6 and n=19). Scatter plots display data from individual mice with black lines showing median values. * $P<0.05$, ** $P<0.01$ by 2-tailed Mann-Whitney *U* test.

Autoantibody	Ctrl	CD11c-*Tlr7*$^{\Delta}$	p-value	Ctrl	B-*Tlr7*$^{\Delta}$	p-value	*Tlr9*$^{-/-}$	B-*Tlr7*$^{\Delta}$ *Tlr9*$^{-/-}$	p-value
Anti-Sm	1367.00 ± 452.90	536.90 ± 357.40	0.0037	1382.00± 513.60	0.10 ± 0.00	<0.0001	1995.00 ± 860.80	7.27 ± 7.17	<0.0001
Anti-RNA	87.74 ± 18.68	39.80 ± 12.14	0.0140	22.80 ± 7.53	3.80 ± 1.34	<0.0001	125.40 ± 24.34	35.81 ± 13.77	0.0002
Anti-nucleosome	2510.00 ± 926.0	3485.00 ± 728.50	0.5796	2288.00 ± 511.20	1997.00 ± 462.40	0.8416	5.07 ± 1.70	1.62 ± 0.64	0.0213

Supplemental Table 1. Autoantibody titers of MRL/lpr serum from indicated strains. Data represent mean ± SEM. Gray boxes indicate statistically significant differences by 2-tailed (CD11c-*Tlr7*$^{\Delta}$ and B-*Tlr7*$^{\Delta}$ cohorts) or 1-tailed (B-*Tlr7*$^{\Delta}$ *Tlr9*$^{-/-}$) Mann-Whitney *U* test.

Cell Type	Ctrl	CD11c-*Tlr7*$^{\Delta}$	Ctrl	B-*Tlr7*$^{\Delta}$	B-*Tlr7*$^{+/+}$	B-*Tlr7*$^{-/-}$	*TLR9*$^{-/-}$	B-*Tlr7*$^{\Delta}$ *Tlr9*$^{-/-}$
B cells	16.36 ± 1.87	17.35 ± 1.57	11.77 ± 1.21	10.47 ± 0.98	8.42 ± 1.29	10.78 ± 1.47	10.54 ± 1.80	16.93 ± 1.72
Marginal Zone (% of B cells)	32.48 ± 2.25	33.15 ± 2.73	40.96 ± 3.11	46.23 ± 2.50	10.20 ± 1.76	14.47 ± 1.54	21.92 ± 2.23	32.43 ± 1.84
Follicular (% of B cells)	35.11 ± 3.95	33.02 ± 2.57	25.85 ± 2.04	27.68 ± 2.22	6.18 ± 0.83	10.20 ± 1.76	38.86 ± 2.43	38.60 ± 1.77
$CD11b^+$ $CD11c^+$ B cells (% of B cells)	2.21 ± 0.23	2.56 ± 0.28	6.28 ± 0.69	6.25 ± 0.72	3.33 ± 0.40	4.46 ± 0.56	3.05 ± 0.32	3.07 ± 0.29
Plasmablasts (% of $TCR\beta^-$)	2.18 ± 0.34	1.51 ± 0.17	1.85 ± 0.44	1.79 ± 0.47	2.26 ± 0.29	2.56 ± 0.33	2.16 ± 0.39	2.05 ± 0.30
cDCs (% of $CD19^-$)	0.54 ± 0.06	0.41 ± 0.04	1.44 ± 0.25	1.19 ± 0.18	1.59 ± 0.15	1.07 ± 0.10	0.50 ± 0.07	0.68 ± 0.05
pDCs (% of $CD19^-$)	0.25 ± 0.03	0.22 ± 0.02	0.15 ± 0.02	0.16 ± 0.02	0.36 ± 0.03	0.36 ± 0.04	0.20 ± 0.03	0.28 ± 0.02
T cells	49.54 ± 2.76	41.45 ± 3.16	46.16 ± 3.23	45.68 ± 3.14	37.15 ± 1.66	35.85 ± 1.40	49.64 ± 3.86	45.81 ± 2.19
CD4 T cells (% of T cells)	24.64 ± 1.40	30.52 ± 1.88	30.28 ± 2.03	32.04 ± 2.04	57.74 ± 0.90	56.31 ± 1.71	25.98 ± 3.01	28.91 ± 1.44
CD4 naïve T cells (% of CD4 T cells)	4.49 ± 0.47	4.24 ± 0.44	5.64 ± 0.93	6.43 ± 1.00	3.29 ± 0.37	3.94 ± 0.45	2.82 ± 0.29	4.11 ± 0.32
CD8 T cells (% of T cells)	22.31 ± 1.21	20.58 ± 1.03	19.94 ± 1.16	21.70 ± 1.23	21.47 ± 0.87	20.66 ± 0.71	14.81 ± 1.78	23.16 ± 1.43
CD8 naïve T cells (% of CD8 T cells)	11.76 ± 0.79	12.20 ± 0.92	10.05 ± 0.62	10.65 ± 0.63	11.46 ± 1.48	12.57 ± 1.41	6.30 ± 0.55	8.56 ± 0.48
Double negative T cells (% of T cells)	50.48 ± 2.08	45.57 ± 1.94	46.69 ± 2.25	43.56 ± 2.00	16.01 ± 0.78	18.55 ± 1.56	52.66 ± 4.55	43.86 ± 2.57
Macrophages (% of $CD11b^+$ $CD19^-$)	8.91 ± 0.78	8.05 ± 1.06	7.82 ± 1.01	9.57 ± 1.26	5.80 ± 0.93	4.75 ± 0.90	9.74 ± 1.31	9.82 ± 0.75
Monocytes (% of $CD11b^+$ $CD19^-$)	12.12 ±1.22	14.14 ± 1.66	7.47 ± 0.91	7.41 ± 0.88	16.12 ± 0.92	15.69 ± 0.91	8.99 ± 1.05	12.08 ± 0.97
Neutrophils (% of $CD11b^+$ $CD19^-$)	22.33 ± 3.50	34.53 ± 4.24	33.62 ± 3.36	31.03 ± 3.17	51.77 ± 2.82	44.60 ± 4.60	37.76 ± 3.06	39.52 ± 2.26

Supplemental Table 2. Percent cell compositions of MRL/lpr spleens from indicated strains. Data represent mean ± SEM. Gray boxes indicate statistically significant differences by 2-tailed (CD11c-*Tlr7*$^{\Delta}$ and B-*Tlr7*$^{\Delta}$ cohorts) or 1-tailed (B-*Tlr7*$^{-/-}$ and B-*Tlr7*$^{\Delta}$ *Tlr9*$^{-/-}$ cohorts) Mann-Whitney *U* test.

Clinical Parameter	Sex	Ctrl	CD11c-*Tlr7*$^{\Delta}$	p-value	Ctrl	B-*Tlr7*$^{\Delta}$	p-value	Ctrl	B-*Tlr7*$^{-/-}$	p-value	*Tlr9*$^{-/-}$	B-*Tlr7*$^{\Delta}$ *Tlr9*$^{-/-}$	p-value
Proteinuria	All	2.56 ± 0.28	2.52 ± 0.25	0.9232	2.73 ± 0.19	1.92 ± 0.17	.0034	2.29 ± 0.19	1.94 ± 0.18	0.1143	2.63 ± 0.25	1.64 ± 0.21	0.0014
	Male	2.33 ± 0.49	2.70 ± 0.28	0.5366	2.93 ± 0.23	2.03 ± 0.25	.0014	1.96 ± 0.14	2.10 ± 0.19	0.2632	**2.92** ± **0.45**	**2.00** ± **0.32**	**0.0781**
	Female	2.67 ± 0.35	2.40 ± 0.38	0.6358	**2.50** ± **0.32**	**1.79** ± **0.24**	**.0643**	2.58 ± 0.30	1.81 ± 0.29	0.0316	2.56 ± 0.32	1.25 ± 0.25	0.0011
Glomerulonephritis	All	2.33 ± 0.14	2.34 ± 0.11	0.7041	2.43 ± 0.23	2.16 ± 0.21	.2137	**3.19** ± **0.32**	**2.57** ± **0.25**	**0.0663**	3.39 ± 0.38	1.92 ± 0.21	0.0001
	Male	2.20 ± 0.13	2.21 ± 0.23	0.7545	2.40 ± 0.34	2.18 ± 0.36	.4891	2.69 ± 0.38	2.35 ± 0.39	0.2449	4.00 ± 0.68	1.79 ± 0.29	0.0009
	Female	2.43 ± 0.22	2.44 ± 0.10	0.4412	2.47 ± 0.31	2.14 ± 0.21	.6821	**3.62** ± **0.48**	**2.55** ± **0.30**	**0.0594**	3.18 ± 0.45	2.06 ± 0.30	0.0204
Interstitial Nephritis	All	1.50 ± 0.12	1.61 ± 0.13	0.6271	1.37 ± 0.11	1.36 ± 0.09	.4650	1.40 ± 0.16	1.67 ± 0.18	0.1175	1.44 ± 0.18	0.69 ± 0.09	0.0001
	Male	1.45 ± 0.16	1.08 ± 0.14	0.1136	1.25 ± 0.15	1.27± 0.16	.9698	1.00 ± 0.12	1.40 ± 0.23	0.0972	1.75 ± 0.28	0.70 ± 0.13	0.0003
	Female	**1.54** ± **0.19**	**2.00** ± **0.14**	**0.0693**	1.50 ± 0.17	1.46 ± 0.08	.7661	1.73 ± 0.24	1.89 ± 0.25	0.3562	1.32 ± 0.22	0.65 ± 0.11	0.0052
Spleen Weight	All	0.54 ± 0.04	0.49 ± 0.04	0.3382	0.57 ± 0.03	0.50 ± 0.04	.1532	0.23 ± 0.02	0.22 ± 0.01	0.3541	0.63 ± 0.06	0.47 ± 0.03	0.0056
	Male	0.45 ± 0.07	0.45 ± 0.07	0.7713	0.49 ± 0.03	0.41 ± 0.04	.1035	0.19 ± 0.01	0.19 ± 0.02	0.3523	**0.72** ± **0.21**	**0.40** ± **0.03**	**0.0674**
	Female	0.61 ± 0.05	0.52 ± 0.04	0.1536	0.66 ± 0.71	0.61 ± 0.05	.4043	0.27 ± 0.03	0.25± 0.02	0.2577	0.60 ± 0.04	0.55 ± 0.04	0.3558
LN Weight	All	0.53 ± 0.03	0.53 ± 0.03	0.5601	0.47 ± 0.03	0.43 ± 0.04	.2426	0.23 ± 0.06	0.23± 0.07	0.3194	0.48 ± 0.04	0.46 ± 0.03	0.2742
	Male	0.51 ± 0.03	0.50 ± 0.05	0.5824	0.45 ± 0.03	0.32 ± 0.04	.0157	00.24 ± 0.10	0.22 ± 0.11	0.1618	0.46 ± 0.09	0.46 ± 0.03	0.3722
	Female	0.55 ± 0.04	0.56 ± 0.04	0.9554	0.50 ± 0.04	0.56 ± 0.06	.4743	0.22 ± 0.07	0.24 ± 0.08	0.4259	0.48 ± 0.04	0.45 ± 0.04	0.2338
Dermatitis	All	1.69 ± 0.33	1.56 ± 0.36	0.8025	2.43 ± 0.27	1.84 ± 0.32	.0961	0.43 ± 0.19	0.77 ± 0.30	0.2741	1.67 ± 0.36	1.26 ± 0.26	0.2013
	Male	2.17 ± 0.69	2.50 ± 0.61	0.6924	3.24 ± 0.33	2.22 ± 0.45	.0740	0.27 ± 0.23	0.50 ± 0.40	0.7243	2.17 ± 0.71	1.60 ± 0.41	0.2887
	Female	1.46 ± 0.37	0.89 ± 0.36	0.3898	1.47 ± 0.32	1.38 ± 0.43	.4537	0.56 ± 0.29	0.98 ± 0.44	0.6205	1.48 ± 0.42	0.81 ± 0.25	0.1423

Supplemental Table 3. Clinical data of MRL/lpr mice from indicated strains. Data represent mean ± SEM. Gray boxes indicate statistically significant differences and bold text indicates data trending towards significance by 2-tailed (CD11c-*Tlr7*$^{\Delta}$ and B-*Tlr7*$^{\Delta}$ cohorts) or 1-tailed (B-*Tlr7*$^{-/-}$ and B-*Tlr7*$^{\Delta}$ *Tlr9*$^{-/-}$) Mann-Whitney *U* test.
